# Colorectal Mucus Binds DC-SIGN and Inhibits HIV-1 *Trans*-Infection of CD4^+^ T-Lymphocytes

**DOI:** 10.1371/journal.pone.0122020

**Published:** 2015-03-20

**Authors:** Martijn J. Stax, Emily E. I. M. Mouser, Thijs van Montfort, Rogier W. Sanders, Henry J. C. de Vries, Henk L. Dekker, Carolina Herrera, Dave Speijer, Georgios Pollakis, William A. Paxton

**Affiliations:** 1 Laboratory of Experimental Virology, Department of Medical Microbiology, Centre for Infection and Immunity, Academic Medical Center, University of Amsterdam, Amsterdam, the Netherlands; 2 Department of Microbiology and Immunology, Weill Medical College of Cornell University, New York, United States of America; 3 Department of Dermatology, Medical Centre of the University of Amsterdam, Amsterdam, The Netherlands; 4 Mass Spectrometry of Biomacromolecules, Swammerdam Institute for Life Sciences, University of Amsterdam, Amsterdam, the Netherlands; 5 Division of Infectious Diseases, Faculty of Medicine, Imperial College, London, United Kingdom; 6 Department of Medical Biochemistry, Medical Centre of the University of Amsterdam, Amsterdam, the Netherlands; 7 Department of Clinical Infection, Microbiology and Immunology, Institute of Infection and Global Health, University of Liverpool, Liverpool, United Kingdom; 8 STI outpatient clinic, Cluster Infectious Diseases, Public Health Service Amsterdam and Centre for Infections and Immunity Amsterdam, Academic Medical Center, University of Amsterdam, Amsterdam, the Netherlands; German Primate Center, GERMANY

## Abstract

Bodily secretions, including breast milk and semen, contain factors that modulate HIV-1 infection. Since anal intercourse caries one of the highest risks for HIV-1 transmission, our aim was to determine whether colorectal mucus (CM) also contains factors interfering with HIV-1 infection and replication. CM from a number of individuals was collected and tested for the capacity to bind DC-SIGN and inhibit HIV-1 *cis*- or *trans*-infection of CD4^+^ T-lymphocytes. To this end, a DC-SIGN binding ELISA, a gp140 trimer competition ELISA and HIV-1 capture/ transfer assays were utilized. Subsequently we aimed to identify the DC-SIGN binding component through biochemical characterization and mass spectrometry analysis. CM was shown to bind DC-SIGN and competes with HIV-1 gp140 trimer for binding. Pre-incubation of Raji-DC-SIGN cells or immature dendritic cells (iDCs) with CM potently inhibits DC-SIGN mediated *trans*-infection of CD4^+^ T-lymphocytes with CCR5 and CXCR4 using HIV-1 strains, while no effect on direct infection is observed. Preliminary biochemical characterization demonstrates that the component seems to be large (>100kDa), heat and proteinase K resistant, binds in a α1–3 mannose independent manner and is highly variant between individuals. Immunoprecipitation using DC-SIGN-Fc coated agarose beads followed by mass spectrometry indicated lactoferrin (fragments) and its receptor (intelectin-1) as candidates. Using ELISA we showed that lactoferrin levels within CM correlate with DC-SIGN binding capacity. In conclusion, CM can bind the C-type lectin DC-SIGN and block HIV-1 *trans*-infection of both CCR5 and CXCR4 using HIV-1 strains. Furthermore, our data indicate that lactoferrin is a DC-SIGN binding component of CM. These results indicate that CM has the potential to interfere with pathogen transmission and modulate immune responses at the colorectal mucosa.

## Introduction

Human immunodeficiency virus type 1 (HIV-1) affects millions of people worldwide despite relatively low transmission rates. Sexual contact is the major route of transmission, yet the risk of transmission is predicted to be 10 times higher when comparing receptive anal intercourse with vaginal intercourse [1]. This may be explained by differences of the epithelium as well as the high number of activated CD4^+^ T-lymphocytes typically found in the gut [2]. Additionally, bodily secretions present at the mucosal surfaces may also play an important role in HIV-1 transmission.

In both routes, HIV-1 is introduced via semen and successful transmission requires the virus to cross a mucosal barrier, either through ruptures, transcytosis or dendritic cell (DC) uptake. Disruption of the mucosal layer can occur during intercourse or result from infections with pathogens such as *Schistosomes*, which have been associated with enhanced HIV-1 transmission [3,4]. However, much emphasis has been placed on *trans*-infection, a mechanism where DCs capture HIV-1 through C-type lectins, mainly Dendritic Cell-Specific Intracellular adhesion molecule-3-Grabbing Non-integrin (DC-SIGN), and transfer the virus to CD4^+^ T-lymphocytes. DCs found below mucosal surfaces can form dendrites which protrude through the epithelial barrier and thereby may facilitate HIV-1 *trans*-infection or where such cells can be exposed to virus through tears and breaches in the mucosal layer [5,6].

DC-SIGN binds mannosylated as well as fucosylated glycans and is thus able to bind an array of pathogens [7,8]. The precise role of DC-SIGN in the infection by these pathogens is unknown. However, studies have indicated that DC-SIGN aids the formation of DC-T-cell synapses which may explain enhanced HIV-1 transmission. Furthermore, it has been shown that DC-SIGN can cross-talk with toll like receptors (TLRs), thereby influencing immune responses generated [8].

Recently a number of host glycoproteins, bile-salt stimulated lipase (BSSL) and mucin (MUC) 1 from human milk as well as MUC6 and clusterin from seminal plasma have been shown to bind DC-SIGN and thereby interfere with HIV-1 capture and transfer to CD4^+^ T-lymphocytes [9–13]. Similarly, a still unidentified molecule in cervical vaginal lavage fluid (CVL) has been described with the same property [14]. Strikingly, the binding capacity of molecules that associate with DC-SIGN and inhibit HIV-1 *trans*-infection, including BSSL, varies between individuals [15]. These studies suggest that the mucosal surface microenvironment may influence the risk of HIV-1 transmission. Furthermore, any strategy aimed at curtailing HIV-1 transmission, including vaccines and microbicides, will have to take into account the presence of such molecules and their activities. Our aim was to determine whether molecules in colorectal mucus (CM) interfere with HIV-1 infection. We found that CM does have a DC-SIGN binding component, which blocks HIV-1 *trans*-infection and identified human lactoferrin from CM as being a molecule with such binding activity.

## Materials and Methods

### CM collection and processing

Mucus was collected by gentle washing with small volumes of PBS of surgically-resected colorectal tissue from HIV-1 negative patients undergoing rectocele repair and colectomy for colorectal cancer (n = 2). CM was collected from healthy tissue located approximately 10 to 15 cm from the tumour. The procedure was performed at St George’s Hospital, London, UK with signed informed consent from the patients [16]. Cells in CM were removed by centrifugation, 30min at 16,000xg. The cell-free supernatant was partially sterilized by passing through a 0.2μm filter. Additional CM samples were collected from male visitors of a STI outpatient clinic (n = 21). They were screened on anal STIs under anoscopic vision and simultaneously, using Dacron swabs, CM was collected from the mucosa. The mean age was 36 years (range 21–63), all subjects tested negative for HIV-1 and showed no signs of sexually transmitted infections. The Ethical committee of the Academic Medical Centre exempted the collection from full review because the lack of additional discomfort. These samples were incubated for 2h at 37°C 1000rpm and subsequently centrifuged 5min at 13,000xg. Next, the Q-tip was inverted and the sample was centrifuged for 1h at 20,000rpm. The collection and research with human colorectal mucus complied with all relevant federal guidelines and institutional board policies.

### ELISA

The DC-SIGN binding ELISA was performed as described [13]. In short, the component of interest was coated on an ELISA plate in 0.2M NaHCO_3_ (pH9.2). After o/n incubation at 4°C the plate was blocked with TSM 5% BSA after which 333ng/ml DC-SIGN-Fc (R&D systems) was added. Subsequently, DC-SIGN was detected by a secondary goat-anti-human-Fc HRP labelled antibody (Jackson Immunology) (1:1000) using standard ELISA procedures. The same set up was used for detecting lactoferrin and intelectin-1 in CM, only instead of DC-SIGN-Fc, 333ng/ml polyclonal anti-lactoferrin (ab15811, Abcam) and 333ng/ml anti-intelectin-1 (ab118232, Abcam) was used. In the DC-SIGN blocking ELISA, 10μg/ml anti-HIV-1 gp120 antibody, D7324 (Aalto BioReagents Ltd) in 0.1M NaHCO_3_ (pH8.6) was coated on an ELISA plate. After overnight incubation at 4°C the plate was blocked with TSM 5%BSA after which trimeric HIV-1 gp140 (JR-FL SOSIP.R6-IZ-D7324) was added to the plate and which has been previously described [17,18]. Meanwhile 333ng/ml DC-SIGN-Fc (R&D systems) was pre-incubated with the component of interest. Subsequently this mixture was added to the gp140 coated plate and DC-SIGN binding was detected by a secondary HRP labelled goat-anti-human-Fc antibody (Jackson Immunology) (1:1000) using standard ELISA procedures. A more detailed description can be found [17]. The Capsid p24 ELISA was performed as standard [10]. Briefly, culture supernatant was added to a sheep anti-p24-specific antibody (Aalto Bio Reagents Ltd.) (10μg/ml) coated ELISA plate. Subsequently, mouse anti-HIV-1-p24 alkaline phosphatase conjugate antibody (Aalto Bio Reagents Ltd.) (4ng/ml) was used as the secondary antibody. For development, Lumi-phos plus (Lumigen Inc.) was used according to the manufacturer's protocol and as a standard curve a serial dilution of *Escherichia coli*-expressed recombinant HIV-1-p24 (Aalto Bio Reagents Ltd.) was analyzed.

### Cells

TZM-bl cells were cultured in DMEM (Invitrogen) containing 10% FCS, MEM non-essential amino acids (0.1mM, Invitrogen), penicillin and streptavidin (pen/strep), both 100U/ml. Raji DC-SIGN cells were cultured in RPMI 1640 (Invitrogen) supplemented with 10% FCS, 100U/ml pen/ strep. The peripheral blood mononuclear cells (PBMC) were isolated from buffy coats (Sanquin) of three healthy CCR5 wild-type homozygous donors using ficoll-hypaque density centrifugation. The cells were pooled and kept at −150°C until required. After thawing PBMCs were cultured in RPMI supplemented with 10% FCS, 100U/ml pen/strep and 100U/ml IL2. 3μg/ml phytohemaglutinin was used to activate the cells and CD4^+^ T-lymphocytes were enriched by removing the CD8^+^ cells using CD8 dynabeads (Life Technologies) according to manufacturer’s protocol. Monocytes were isolated from buffy coats by ficoll-hypaque density centrifugation followed by selection for CD14^+^ cells via MACS (Miltenyi Biotec). Monocytes were cultured in RPMI containing 10% FCS supplemented with 500U/ml IL4 and 800U/ml GM-CSF (Schering-Plough) for 6 days which differentiated them into iDCs [19].

### Viruses

Replication-competent HIV-1 subtype B NSI-18 (R5) and subtype B LAI (X4) were passaged on CD4^+^ T-lymphocytes [9]. NSI-18 is a primary isolate obtained from an individual from the Amsterdam cohort studies of Gay men and which utilises CCR5 as its coreceptor and LAI represents a molecular clone isolated from an HIV-1 patient and which utilises CXCR4. For each batch the tissue culture infectious dose (TCID_50_) was determined by limiting dilutions on CD4^+^ T-lymphocytes according to the Reed and Muench method, previously described [20]

### Direct infection

Different CM dilutions were pre-incubated, 30min. on ice, with NSI-18 or LAI (5ng/ml p24) in DMEM supplemented with 10% FCS, 100U/ml pen/strep and 40μg/ml (end concentration) DEAE-dextran (Sigma). Subsequently, this mixture was added to TZM-bl cells (70–80% confluent) that were washed with PBS. Two days post infection the cells were washed again with PBS after which they were lysed with Reporter Lysis Buffer (Promega). After at least 30min. at −80°C the luciferase activity was measured on the Glomax luminometer (Turner BioSystems) using the Luciferase Assay kit (Promega) according to manufacturer’s protocol. Direct infection of CD4^+^ T-lymphocytes was determined by pre-incubating these cells with different dilutions of CM for 30min after which LAI (1000 TCID_50_) was added. Viral growth was monitored by measuring capsid p24 levels in culture supernatants using ELISA.

### DC-SIGN mediated *trans*-infection and FACS analysis

In Raji DC-SIGN mediated capture/transfer experiments Raji DC-SIGN cells (5x10^4^ cells/well) were pre-incubated with CM for 30min after which 500 TCID_50_/ml HIV-1 isolate NSI-18 or LAI was added. After 1h the cells were washed thoroughly and co-cultured with 2x10^5^ CD4^+^ T-lymphocytes/well. Viral outgrowth was measured by capsid p24 ELISA. HIV-1 capture/transfer by iDCs and FACS analysis of the infection was performed as described [27] with minor alterations. Briefly, 1x10^5^ iDCs were pre-incubated with CM for 30min, 1x10^3^ TCID_50_/ml virus (end concentration) was added, and after 2h the iDCs were washed and co-cultured with 2x10^5^ CD4^+^ T-lymphocytes. After 48h the medium was replaced by fresh RPMI containing rIL-2 (2μg/ml) and indinavir (1μM; NIBSC). For FACS analysis the cells were fixed in 3.7% formaldehyde and subsequently permeabilized in PBS containing 1% BSA, 50mM NH_4_Cl and 0.1% saponin (Riedel-deHaën, S060905). Using anti-human CD3-APC (1:100, BD Pharmingen) and anti-CA-p24-FITC (1:200, Coulter Clone) the number of infected cells per 1x10^5^ CD3^+^ T-lymphocytes was determined.

### Biochemical characterization

Size fractionation of CM was performed with YM-30 and YM-100 Microcon centrifugal filter devices (Millipore) according to manufacturer instructions. Heat treated samples were placed in a heat block for 10min at 95°C and proteinase K (Promega) treated samples were incubated for 30–60min at 56°C after which the enzyme was inactivated (10 min at 95°C). The depletion of (α1–3) mannose containing glycans was performed with *Galanthus Nivalis* lectin coated agarose beads (Vector Laboratories) according to the manufacturer’s protocol. In short, the samples were incubated with the beads for 1h at RT after which the beads were removed from the sample via centrifugation.

### Mass spectrometry analysis

To prepare samples for mass spectrometry analysis, CM of 6 DC-SIGN high binding individuals were pooled and incubated on a Protein A/G (Pierce Biotechnology) column overnight. An immunoprecipitationcarboxy using DC-SIGN-Fc (R&D systems) coated agarose beads (Sigma) was performed on the throughput, beads and CM were incubated together O/N. After two wash steps the beads were taken up in DTT containing sample buffer, placed at 99°C for 10min and run on a 4–12% SDS PAGE gel (Novex, Life Technologies). Protein bands of interest were excised, alkylated and subjected to tryptic digestion according to standard protocols. Digests were analysed using a Bruker Ion Trap (Amazon Speed) upon separation by a 30min LC run using a C-18 column controlled by an EasyLC system (Bruker-Protana). Acquired data files were processed by the Bruker Compass DataAnalysis software (version 4.1, build 359) and exported MGF files (Mascot generic) were used to search with Mascot. Search parameters for Mascot were: ESI-trap; charge states: 1+, 2+, 3+; tolerance: 0.3; ms/ms tolerance: 0.4; trypsin; one missed cleavage; fixed modification: carbamidomethylcysteine; variable modification: oxidation of methionine. Probabilistic Mascot scoring evaluates peptide identifications and *p* values of less than 0.05 are considered significant.

This approach resulted in identification of human lactoferrin with a total score of 211 -15% sequence coverage- (in band 2), and 302 -16% coverage- (in band 3), as well as human intelectin-1 (band 4) with a total score of 148 and 18% coverage. Band 2 does not contain higher scoring human proteins than lactoferrin (apart from some immunoglobulin and keratin contaminants); at much lower confidence levels we find the polymeric immunoglobulin receptor (score 66), intelectin-1 (score 46), IgGFc-binding protein (score 39), and Mucin-2 (score 30). Band 3 does not contain higher scoring human proteins than lactoferrin (apart from some immunoglobulin and keratin contaminants); at much lower confidence levels we find IgGFc-binding protein (score 98), Krev interaction trapped protein I (score 48), and the polymeric immunoglobulin receptor (score 43). Band 4 does not contain higher scoring human proteins than intelectin-1 (apart from some immunoglobulin and keratin contaminants); at lower confidence levels we find lactoferrin (score 102), serum albumin (score 44), and IgGFc-binding protein (score 31).

### Statistical analysis

Two tailed unpaired t-tests were performed for data sets except when comparing DC-SIGN binding to CM (OD450) with DC-SIGN binding to gp140 (OD450) where a Spearman’s rank correlation was used. P values <0.05 were considered statistically significant.

## Results

### CM binds DC-SIGN and blocks its interaction with HIV-1 envelope trimer

**Fig 1:**
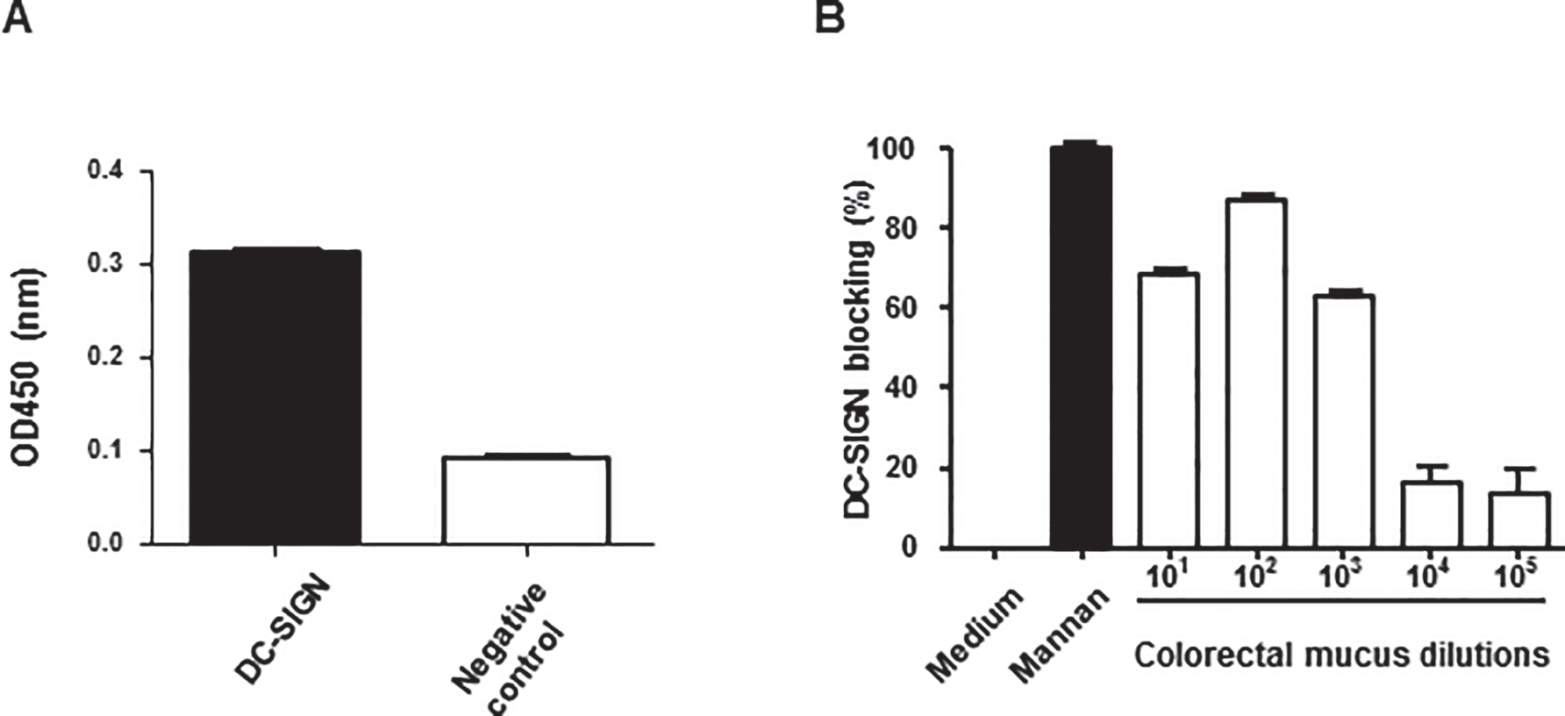
CM binds DC-SIGN thereby preventing gp140 binding. (A) DC-SIGN binding ELISA with 300 fold diluted CM coated on an ELISA plate and DC-SIGN-Fc as detection antibody, demonstrates DC-SIGN-Fc binds CM compared to EGTA treated product (negative control) (p<0.0001). (B) DC-SIGN-Fc was pre-incubated with mannan (positive control) and CM dilutions before being added to a gp140 coated plate. Depicted is the percentage by which DC-SIGN-Fc binding to gp140 is blocked, pre-incubation with mannan was set to 100% blocking and pre-incubation with medium to 0%. Pre-incubating DC-SIGN-Fc with up to a 1000 fold diluted CM inhibits HIV-1 envelope gp140 trimer binding. Data points were performed in triplicate.

Using a DC-SIGN binding ELISA, the ability of CM to bind DC-SIGN was determined. As Ca^2+^ ions are required for specific DC-SIGN binding, incubation of DC-SIGN-Fc in the presence of the calcium chelator EGTA served as a negative control. We observed that CM bound DC-SIGN-Fc in comparison to the EGTA inactivated DC-SIGN-Fc (p<0.0001, Fig. 1A). Likewise, DC-SIGN-Fc also bound HIV-1 trimeric gp140 SOSIP Env, which is inhibited when DC-SIGN-Fc was pre-incubated with up to a 1000 fold diluted CM in the DC-SIGN blocking ELISA (Fig. 1B). Together, these results suggest that CM not only binds to DC-SIGN, but also inhibits DC-SIGN binding to the HIV-1 envelope trimer.

### CM blocks DC-SIGN mediated *trans*-infection

**Fig 2:**
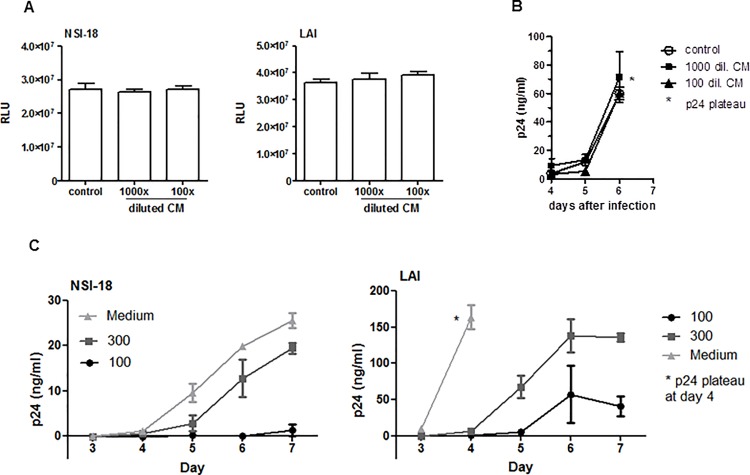
CM does not inhibit HIV-1 direct infection but does *trans*-infection. (A) NSI-18 (R5) or LAI (X4) (5ng/ml p24) was pre-incubated with medium (control) or 100 fold and 1000 fold diluted CM after which the mixture was added to TZM-bl cells. Two days post infection the cells were lysed and the luciferase activity was measured demonstrating that the level of infection was similar whether CM was present or not. (B) CD4^+^ T-lymphocytes were incubated with medium (control) or 100 fold and 1000 fold diluted CM prior to addition of LAI. Viral outgrowth, supernatant p24, was measured over several days, with no difference observed. (C) Raji DC-SIGN cells were incubated with medium (negative control) or 100 fold diluted CM or 300 fold diluted CM prior to addition of either NSI-18 or LAI virus, washed and added to CD4^+^ T-lymphocytes. Viral outgrowth, determined by capsid p24 ELISA, is depicted in Raji DC-SIGN cell—CD4^+^ T lymphocyte co-cultures. For both NSI-18 and LAI inhibition is observed with 100 fold diluted CM and less with 300 fold diluted CM. Data points were performed in triplicate.

We initially investigated whether CM had the capacity to modulate direct infection of HIV-1. Infection of TZM-bl cells was not affected by either 1000 fold or 100 fold dilutions of CM for either NSI-18 (R5) or LAI (X4) HIV-1 (Fig. 2A). Additionally, the same dilutions of CM had no effect on LAI infection and replication in enriched CD4^+^ T-lymphocytes (Fig. 2B). To determine the effect of CM on DC-SIGN-mediated HIV-1 capture and transfer, Raji DC-SIGN cells were incubated with different dilutions of CM (300 and 100 fold), medium or mannan (controls) before addition of HIV-1. Subsequently, these cells were co-cultured with CD4^+^ T-lymphocytes and viral outgrowth was monitored. We observed a clear delay in outgrowth of both NSI-18 and LAI when Raji DC-SIGN cells were pre-treated with 100 fold diluted CM and a less pronounced delay when CM was diluted 300 fold (Fig. 2C). Since CM had no effect on direct infection of CD4^+^ T-lymphocytes the observed delay in viral outgrowth can be attributed to inefficient HIV-1 capture and transfer in the presence of CM.

**Fig 3:**
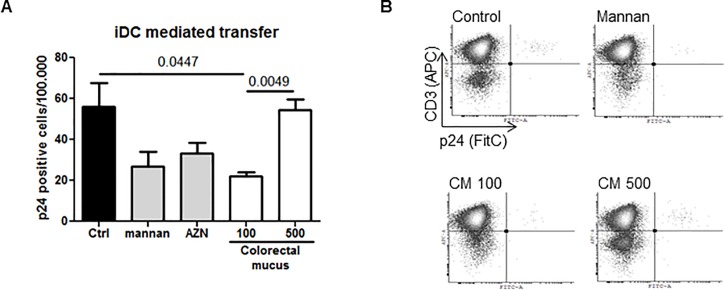
CM inhibits HIV-1 *trans*-infection by iDCs. (A) iDCs were pre-incubated with 100 fold diluted CM, 20μg/ml AZN-D1 (DC-SIGN blocking antibody), 50μg/ml mannan or medium (control) before addition of LAI. Using flow cytometry viral outgrowth in iDC – CD4 T-lymphocyte co-cultures was measured by intracellular staining for p24. Depicted is the number of p24^+^ cells per 1x10^5^ CD3^+^ T cells. (B) Shown are representative dot plots of the data depict in (A). Data points were performed in triplicate.

Next, we repeated these experiments using the physiologically more relevant immature monocyte derived DCs (iDC). These were pre-incubated with CM or medium, 20μg/ml DC-SIGN blocking antibody AZN or 50μg/ml mannan which served as controls. The result demonstrates that 100 fold diluted CM inhibits viral capture and transfer of LAI by iDCs and this effect was lost when CM was diluted 500 times (Fig. 3A). A representation of the assay results obtained is shown (Fig. 3B). These results indicate that CM can efficiently inhibit *trans*-infection of HIV-1 by iDCs.

### Biochemical characterization of the DC-SIGN binding component of CM

**Fig 4:**
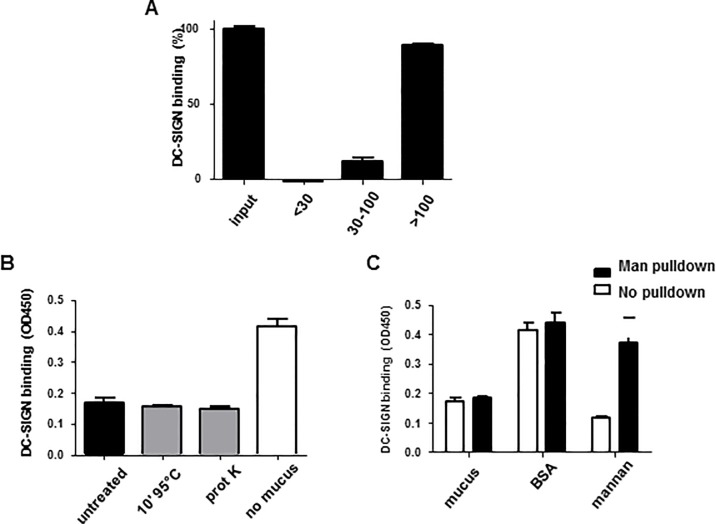
Biochemical analysis of the DC-SIGN binding component in CM. (A) Unfractionated CM (input) or <30, 30–100 or >100kDa CM fractions were coated to an ELISA plate and DC-SIGN-Fc binding was determined. Binding of unfractionated CM is set to 100% and binding of the fractions is expressed as a relative percentage. The <30kDa fraction shows no DC-SIGN binding, between 30–100kDa shows limited binding whilst >100kDa shows stronger binding. (B) DC-SIGN-Fc was incubated with untreated, heated (10 min at 95°C), proteinase K treated CM or medium (negative control) prior to addition to a gp140 coated plate. Compared to the media alone control incubating DC-SIGN-Fc with treated or untreated CM led to similar reductions in gp140 binding. (C) DC-SIGN-Fc was incubated with CM, BSA or mannan prior to addition to a gp140 coated plate. Untreated, both CM and mannan inhibit DC-SIGN-Fc from binding gp140 compared to BSA (negative control). Depletion of mannose structures from CM, BSA and mannan by a pull-down with *Galanthus Nivalis* lectin does not alter the DC-SIGN-Fc binding capacity of CM while mannan loses its ability to prevent gp140 binding by DC-SIGN-Fc. Data points were performed in triplicate.

Initially CM was size-fractionated using 30- and 100-kDa centrifugal filter devices. Utilizing the DC-SIGN binding ELISA we found that the fraction above 100 kDa contained factor(s) with strong DC-SIGN binding properties whereas weak binding was observed in the 30–100 kDa fraction and the below 30 kDa fraction did not bind DC-SIGN-Fc (Fig. 4A). Non-fractionated CM (input control) and negative (EGTA) controls were included in the DC-SIGN binding ELISA for all tested samples. Determined by the DC-SIGN blocking ELISA, CMs ability to prevent gp140 binding to DC-SIGN was not affected by either heat (96°C), proteinase K treatment (Fig. 4B) or the depletion of α1–3 linked mannose glycans (Fig. 4C).

### DC-SIGN binding of CM varies between individuals

**Fig 5:**
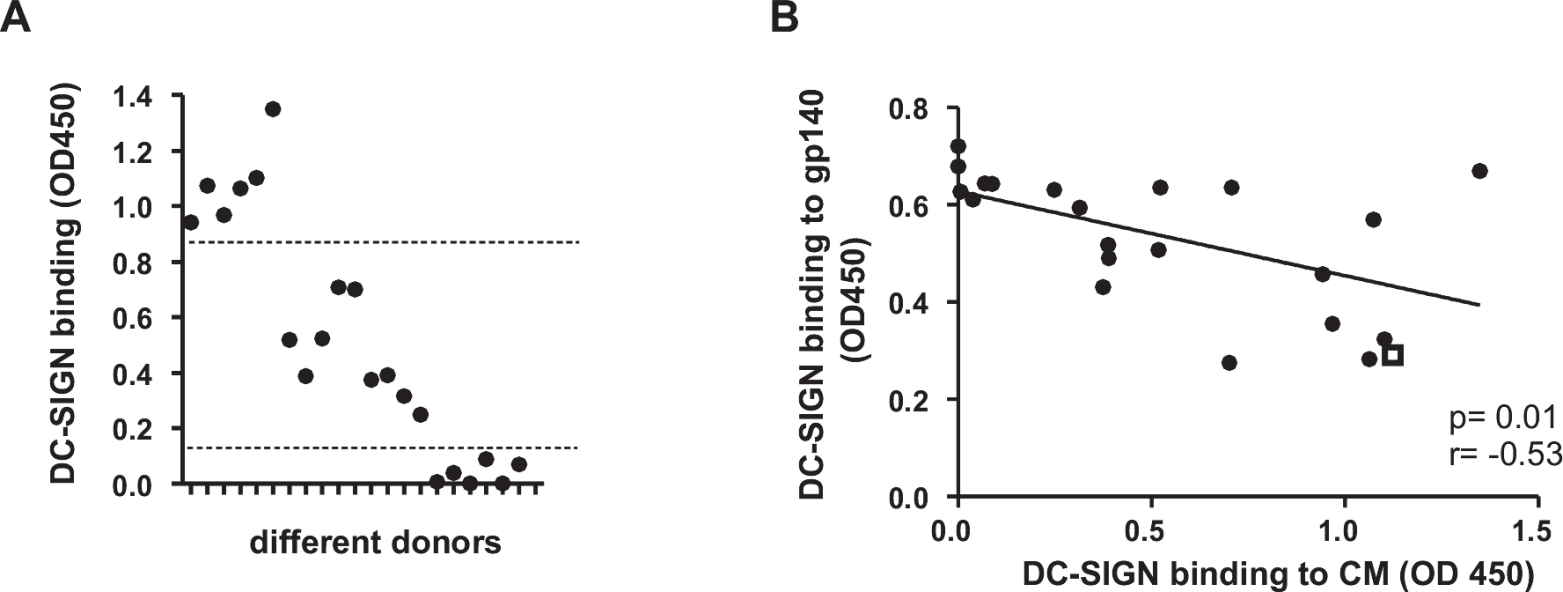
DC-SIGN binding capacity of CM varies greatly between individuals. (A) Serial dilutions of the 21 CM samples were coated onto an ELISA plate and the end-point dilution showing binding of DC-SIGN-Fc is depicted. For all samples maximal binding was achieved when 11.7μg/ml CM was coated. A large variation between donors was observed ranging from high to no DC-SIGN binding and where the samples can be divided into three groups as indicated with dotted lines (B). The ability of samples to prevent DC-SIGN-Fc binding to trimeric gp140 was determined with a blocking ELISA. 11.7μg/ml CM was pre-incubated with DC-SIGN-Fc before addition to a gp140 coated plate. Next, DC-SIGN binding to gp140 was correlated with the OD found in the DC-SIGN binding ELISA where 11.7μg/ml CM was coated (P<0.01). As a control 5μg/ml mannan was included, depicted as an open square.

To evaluate donor variation, 21 additional samples were analyzed using a serial dilution from 750μg/ml to 11.4ng/ml protein input in the DC-SIGN binding ELISA. Four samples did not bind DC-SIGN, whereas the remaining samples reached a binding plateau at 11.7μg/ml input. We observed clear differences in the DC-SIGN binding capacity of CM between individuals which could be divided in high, intermediate and low/no DC-SIGN binding (Fig. 5A). To determine whether the DC-SIGN binding capacity correlates to the ability to prevent gp140 binding, we plotted the DC-SIGN binding capacity versus the gp140 binding by DC-SIGN pre-incubated with 11.7μg/ml CM (Fig. 5B). Results show a clear correlation (p = 0.01) and, interestingly, a few CM samples were comparable in their DC-SIGN binding and gp140 blocking capacity to the positive control mannan (open square, Fig. 5B).

### Mass spectrometry identifies lactoferrin as a DC-SIGN binding component in CM

**Fig 6:**
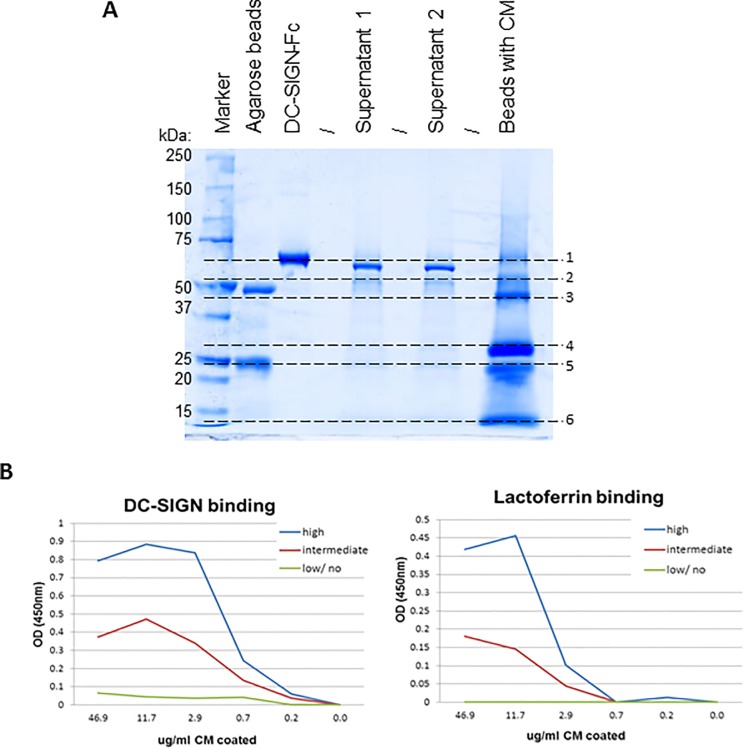
Mass spectrometry indicates that human CM lactoferrin binds DC-SIGN. (A) 4–12% SDS PAGE gel loaded (from left to right) with a 250kD protein marker, agarose beads, DC-SIGN-Fc, the supernatant from the first wash, supernatant from the second wash, and the DC-SIGN-Fc coated agarose beads loaded with the DC-SIGN binding component from CM. Band #2, 3 and 4 potentially contain the DC-SIGN binding component of CM. Ion trap mass spectrometry of in gel digests identified human lactoferrin fragments (highly abundant in band #3) and immunoglobulins in all three bands and intelectin-1 in band #4 (with trace amounts in the other bands). (B) CM representing a high DC-SIGN binder, an intermediate DC-SIGN binder and a low/no DC-SIGN binder were coated on an ELISA plate and were tested for DC-SIGN and lactoferrin binding. The first graph (left) confirms the DC-SIGN binding status while the second graph (right) shows the binding capacity of polyclonal anti-lactoferrin, which is high for CM from a DC-SIGN high binder, intermediate for an intermediate DC-SIGN binder and not present in CM from a low/no DC-SIGN binder.

In order to identify the DC-SIGN binding component within CM we performed an immunoprecipitation on pooled CM from 6 samples showing high DC-SIGN binding and which had been depleted of antibodies, using DC-SIGN-Fc coated agarose beads. Subsequently, the DC-SIGN-Fc coated agarose beads with the component(s) of CM were ran on an SDS PAGE gel resulting in the identification of three bands (# 2, 3 and 4, Fig. 6A). Following tryptic in gel digestion and mass analysis of the resulting peptides, using ion trap mass spectrometry, the proteins human lactoferrin (highly abundant in band #3) and immunoglobulin were identified in all bands, whereas intelectin-1 (lactoferrin receptor) was identified abundantly in band #4 (with trace amounts in the other bands). To confirm lactoferrin as a DC-SIGN binding component in CM, we coated CM from a high, an intermediate and a low/no DC-SIGN binder to an ELISA plate and determined binding of DC-SIGN-Fc, polyclonal anti-lactoferrin and anti-intelectin-1. We found high levels of lactoferrin in CM of a high DC-SIGN binder, intermediate levels in CM of an intermediate DC-SIGN binder and no lactoferrin in CM of a low/no DC- SIGN binder, whilst no intelectin-1 binding was observed (Fig. 6B). Taken together these results indicate that lactoferrin from CM potently binds DC-SIGN.

## Discussion

To gain better insight into mechanisms involved in HIV-1 transmission deciphering the role specific bodily fluids can play in modulating infection is crucial. Here we demonstrate that although CM is unable to protect against direct infection it does block HIV-1 capture and subsequent transfer by both Raji-DC-SIGN cells and iDCs to CD4^+^ T-lymphocytes. Other bodily fluids, such as semen, contain factors able to either inhibit or enhance HIV-1 infection. For example, spermatozoa and Semen Derived Enhancer of Virus Infection (SEVI, small aggregates or fibrils) can bind HIV-1 and promote infection of target cells [21,22], whilst semenogelin-I inhibits direct infection of target cells and MUC6 and clusterin interfere with DC-SIGN mediated *trans*-infection [11,13,23]. Similarly, CVL contains several innate antimicrobials which offer protection against direct infection as well as a glycoprotein that prevents HIV-1 from binding DC-SIGN [14,24]. Thus an additional explanation for the enhanced probability of HIV-1 transmission via anal, as compared to vaginal, intercourse may be the lack of inhibitors in CM that are able to block direct infection.

Analysis of the DC-SIGN binding component in CM revealed it binds DC-SIGN in a α1–3 mannose independent manner, which implies that the factor is likely fucosylated as DC-SIGN recognizes either of these two carbohydrate structures [7]. Mass spectrometry analysis of a DC-SIGN pull down fraction from CM suggested lactoferrin fragment(s) as the (major) DC-SIGN binding component in CM, since they were identified in band 2, very abundantly in 3 (containing 10 peptides, all from the carboxy-terminal half of the protein) and in band 4 of our SDS PAGE gel. Indeed, we find high levels of lactoferrin in CM with high DC-SIGN binding capacity, intermediate levels in CM with intermediate DC-SIGN binding and no lactoferrin when there is low/no DC-SIGN binding, indicating human lactoferrin in CM is able to bind DC-SIGN. Previous studies have indicated that human lactoferrin does not, or only weakly, binds DC-SIGN [25, 26]. However, these studies have been conducted with recombinant lactoferrin. The strength of binding observed with CM may be explained by differences in post-translational modifications, such as glycosylation, or processing which could modulate binding to DC-SIGN. Interestingly, the DC-SIGN binding molecule in CM ended up in the larger than 100kDa fraction upon size fractionation, while the molecular weight of human lactoferrin from milk is approximately 80kDa in size. The actual sizes of the lactoferrin fragments found on the SDS PAGE gel were all well below 75kDa. The sample was however boiled and treated with DTT which destroys any complexes and since intelectin-1, a gut specific lactoferrin receptor, was also identified in the DC-SIGN pull down fraction it is likely lactoferrin and intelectin-1 form a complex which could explain the higher predicted molecular weight. Our results imply that intelectin-1 binding to lactoferrin does not interfere with DC-SIGN binding, as high DC-SIGN binding activity is found in the fraction most likely containing complexes. However, lactoferrin (fragment)-intelectin-1 complex formation does abolish recognition of intelectin-1 by a polyclonal antibody. The fact that we identified DC-SIGN binding of lactoferrin while earlier studies did not [25,26] could also be explained by the fact that the carboxy-terminal fragment of the molecule is involved. This could result from mechanisms such as changes in exposition of the binding site(s) upon fragmentation or intelectin-1 binding leading to improved DC-SIGN binding.

Mass spectrometric analysis revealed no indication for MUC proteins binding DC-SIGN. Given that MUC1 from human milk and MUC6 from seminal plasma are known to inhibit DC-SIGN mediated HIV-1 *trans*-infection one might have expected another member of this family to be expressed in CM and binding DC-SIGN [12,27]. Previously, BSSL from human milk has been demonstrated to bind DC-SIGN and inhibit *trans*-infection, while certain allele combinations are correlated with a lower risk of HIV-1 infection, indicating BSSL potentially protects against transmission [9,10,28]. Furthermore, this molecule is produced in the pancreas and can be released into the duodenum, making it likely that BSSL or smaller digested fragments could be present in CM [29]. However, neither MUC proteins nor BSSL were identified in the DC-SIGN pull-down assay indicating that if these glycoproteins are present, they are only so in limited amounts. Of course we cannot exclude the possibility that the pull down assay preferentially allowed for the capture of human lactoferrin, whereby other DC-SIGN binding molecules were missed.

In conclusion, as with other bodily fluids, CM contains a DC-SIGN binding component with the ability to block HIV-1 *trans*-infection, with human lactoferrin contributing to the binding. These results indicate that CM has the potential to interfere with HIV-1 transmission whilst simultaneously restricting antigen capture at the anal mucosa and potentially skewing mucosal immune responses in the rectum.
